# Shear Bond Strength of Composite Diluted with Composite-Handling Agents on Dentin and Enamel

**DOI:** 10.3390/polym14132665

**Published:** 2022-06-30

**Authors:** Mijoo Kim, Deuk-Won Jo, Shahed Al Khalifah, Bo Yu, Marc Hayashi, Reuben H. Kim

**Affiliations:** 1 Restorative Materials and Applied Dental Research Laboratory, UCLA School of Dentistry, Los Angeles, CA 90095, USA; vagusmj@gmail.com (M.K.); jdw@snubh.org (D.-W.J.); salkhalifah@dentistry.ucla.edu (S.A.K.); boyu@dentistry.ucla.edu (B.Y.); mhayashi@dentistry.ucla.edu (M.H.); 2 Section of Restorative Dentistry, UCLA School of Dentistry, Los Angeles, CA 90095, USA; 3 Department of Prosthodontics, Section of Dentistry, Seoul National University Bundang Hospital, Seongnam 13620, Korea

**Keywords:** dental composite, composite-handling agents, shear bonding strength, wetting resin, sculpturing resin, modeling resin

## Abstract

This in vitro study aimed to examine the shear bond strength of composite on the dentin and enamel substrates when mixed with different composite-handling agents (CHAs). Eighty extracted molars were embedded into acrylic resin and sectioned sagittally. On the prepared specimens, four groups of resin mixtures were bonded onto the enamel or dentin surfaces—composite only, composite mixed with Composite Wetting Resin (CWR), composite mixed with Brush and Sculpt (BS), and composite mixed with Modeling Resin (MR). All groups were prepared by mixing at a 1:1 ratio by weight. Each specimen was subjected to the shear bond strength test. After the test, adhesive or cohesive failures were examined at the fractured sites. Data were analyzed using one-way and two-way analysis of variance (ANOVA) and the Tukey post hoc test. All composite groups mixed with CHAs displayed a reduced shear bond strength on dentin and enamel substrates compared to composite alone (*p* < 0.05). The shear bond strength on dentin decreased in the following order: CWR > BS > MR. A similar pattern was observed on enamel, except that there was no statistically significant difference between BS and MR. Statistically significant interactions between resin mixtures and substrates were found (*p* < 0.001). On the dentin substrate, adhesive failure dominated while adhesive/cohesive failure dominated on the enamel substrate. Conclusions: The shear bonding strength of composite decreases when mixed with CHAs on both dentin and enamel substrates.

## 1. Introduction

Composite resin restorations have become increasingly popular in restorative dentistry due to high esthetic demands and improved mechanical/physical behaviors. Multiple shade options allow for faithful replication of the natural teeth’ translucency, opalescence, and fluorescence [1]. Over the years, a significant improvement in composition, from macrofilled to nanofilled and/or nanohybrid composites, resulted in enhanced overall material properties of composites [2]. Furthermore, the advances in adhesive technologies and minimally invasive operative techniques significantly contributed to the increasing popularity of composite resin restorations [3], leading to better clinical performance and increased longevity.

Despite many signs of progress in the performance of dental composites, the longevity of dental restorations is still limited. Annual failure rates of up to 0–45% have been reported for dental composite restoration in posterior and anterior teeth [4,5,6,7,8]. The primary reasons for composite failure were secondary caries, restoration fracture, and marginal defects [8]. Thus, the key aspect of restoring a tooth for the long-term success is to use the composite resin of clinically acceptable strength without any detrimental factors such as saliva, blood, and hemostatic agents compromising bonding quality [9,10,11] and is to reconstitute the form and function of the damaged tooth without any voids and open margins. Clinically, however, a complete marginal seal with no voids is a challenging task due to the poor handling property of composite; the viscous resin monomers in composites render it sticky to the instruments and difficult to sculpt due to “pull back” during condensation [12,13,14]. 

Many clinicians use low-viscosity materials such as dental adhesives to achieve the ideal placement and margin seal. The technique is to apply the adhesive on the surface of the first composite increments before light curing or on the hand instrument, enabling the easy modeling of the next increment [15]. Dental adhesives reduce the surface tension of sticky resins, enabling easy application of composite resin into the prepared tooth and may decrease the air trapping inside the restorations [15,16], although the manufacturers do not officially recommend the technique. Several dental companies introduced composite-handling agents (CHAs) such as modeling resin or wetting resin to lubricate the hand instruments and prevent composite adhesions. Despite the prevalence of this practice, the mixing of CHAs may adversely impact the physical and surface properties of the composite [13,14,17,18,19]. The CHAs have lower filler content and are presumably weaker than the composite itself; accordingly, it is suggested that a mixture of composite with the handling agents at the margin may dilute the actual composition of the composite and weaken the marginal integrity [20,21]. However, studies to date have focused on the innate properties of the composite when mixed with CHAs, and the investigations on the bonding interface, which is more consequential for the marginal integrity of the restorations, are lacking.

This study aims to examine the shear bond strength of the composite diluted with various CHAs that are commercially available. The null hypothesis of this in vitro study is that the shear bond strength of composite does not change when mixed with CHAs.

## 2. Materials and Methods

### 2.1. Teeth Preparation

Third molars were obtained for the test by the approval of the UCLA institutional review board as Exempt #4 (protocol #14-000270). The teeth were sectioned sagittally using a trim saw diamond blade (Lapcraft Trim Saw; Lapcraft, Powell, OH, USA) and then embedded in self-curing acrylic resin (monomer and polymer resin; Great Lakes Orthodontics, Tonawanda, NY, USA). The sectioned surface was ground utilizing 400- and 600-grit SiC paper for 60 s with water irrigation to simulate a smear layer after tooth preparation. 

### 2.2. Bonding Agent and Composite Resin Application

**Figure 1:**
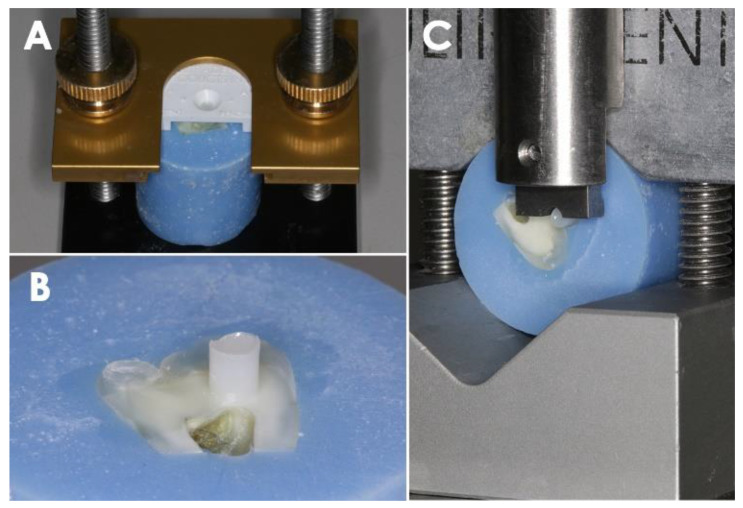
Specimen preparation. (**A**) An acrylic-resin embedded tooth was mounted onto the bonding clamp and bonding mold. (**B**) Composite alone or composite mixed with different CHAs were bonded onto either dentin (or enamel) surface. (**C**) Shear bonding test was then performed.

Eighty teeth were assigned randomly to each of the experimental groups in groups of 20 as follows: Group 1, Filtek Z250 (Comp; 3M, Seefeld, Germany); Group 2, Filtek Z250 mixed with Composite Wetting Resin (CWR; Ultradent, South Jordan, UT, USA); Group 3, Filtek Z250 mixed with Brush and Sculpt (BS; Cosmedent, Chicago, IL, USA); Group 4, Filtek Z250 mixed with Modeling Resin (MR; Bisco, Schaumburg, IL, USA). Each mixture in Group 2–4 was made at a 1:1 ratio by weight for 30 min in a 37 °C water bath without light exposure. Compositions of each handling resin are shown in Table 1. Each prepared tooth was lightly dried for 5 s using an absorbent paper. Scotchbond Universal Adhesive (3M ESPE, Seefeld, Germany) was then applied evenly to each specimen with a microbrush utilizing the self-etch mode. The bonding agent was then cured using a halogen light-curing unit (Variable Intensity Polymerizer; Bisco, Schaumburg, IL, USA; 600 mW/cm^2^) for 10 s according to the manufacturer’s instructions. Composite resin in each group was then packed into a bonding clamp assembled with a mold insert (Model no. 34,224 and 34,228; Ultradent, South Jordan, UT, USA) and light-polymerized for a total of 60 s in 2 mm increments. The specimen was separated from the clamp and confirmed free of air bubbles or interfacial gaps using a light stereomicroscope (SE303R-P; AmScope, Irvine, CA, USA) at 10× magnification (Figure 1A,B).

### 2.3. Shear Bond Strength Test

The specimens were then placed in the test base clamp, and the shear bond strength tests were performed at a crosshead speed of 1 mm/min using the UltraTester (Ultradent, South Jordan, UT, USA) as in Figure 1C. The notched crosshead assembly and the test base clamp (Model no. 34,223; Ultradent, South Jordan, UT, USA) with the specimen were positioned to contact the bonded specimen at the composite and dentin/enamel interface. The shear load was applied until failure occurred. After that, the obtained values of shear bond strength were expressed in megapascals (MPa) by dividing the recorded peak load at failure (N) by the adhesive surface area (mm^2^).

### 2.4. Cohesive and Adhesive Failure Evaluation

The failure modes were evaluated for each sample and classified as an adhesive failure (A) at the dentin–resin interface, a cohesive failure in resin (CR), a cohesive failure in the tooth (CT), and a mixed failure (A + CR or A + CT). Eleven samples in each group were examined to determine failure mode under the stereomicroscope at 30× magnification.

### 2.5. Statistical Analysis

The shear bond strength was described as means ± standard deviations. Statistical analysis was performed using one-way analysis of variance (ANOVA) for different resin mixtures and two-way ANOVA to examine the interaction effects between resin mixtures and substrates. A post hoc Tukey test was performed for multiple comparisons. *p* < 0.05 was considered to be statistically significant. SPSS Statistics version 26.0 (SPSS Inc., Chicago, IL, USA) was used for statistical analysis.

## 3. Results

### 3.1. Shear Bond Test to the Dentin Surface

**Figure 2:**
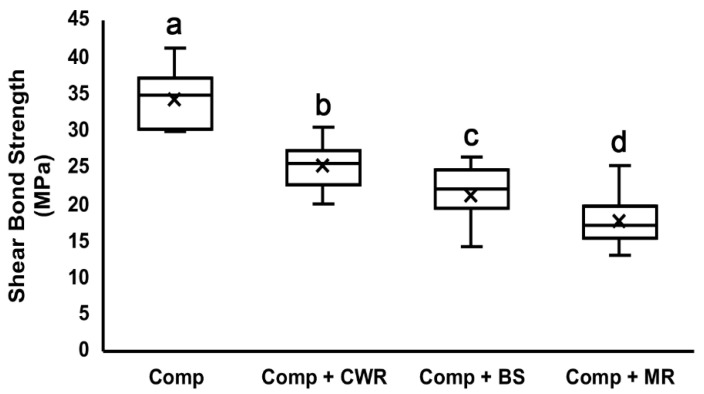
Shear bond test to the dentin surface. Composite mixed without or with different handling agents were bonded onto the dentin surface and subjected to the shear bonding test. Different letters indicate significant differences between groups (*p* < 0.05). The ‘x’ in the box indicates the mean value of each group. Comp = Composite; CWR = Composite Wetting Resin; BS = Brush and Sculpt; and MR = Modeling Resin.

The results of the shear bond test to the dentin surface are shown as means ± standard deviations (Table 2) and as a box plot (Figure 2). All tested CHAs demonstrated significant reductions in shear bond strength. Compared to the composite-only group (34.16 ± 3.65 MPa), the CWR group showed the least but a significant reduction (25.09 ± 3.10 MPa), followed by the BS group (21.24 ± 3.37 MPa) and MR group (17.73 ± 3.26 MPa), which showed the most significant decrease in shear bond strength. Differences in the shear bond strengths are statistically significant amongst all groups (*p* < 0.05).

### 3.2. Shear Bond Test to the Enamel Surface

**Figure 3:**
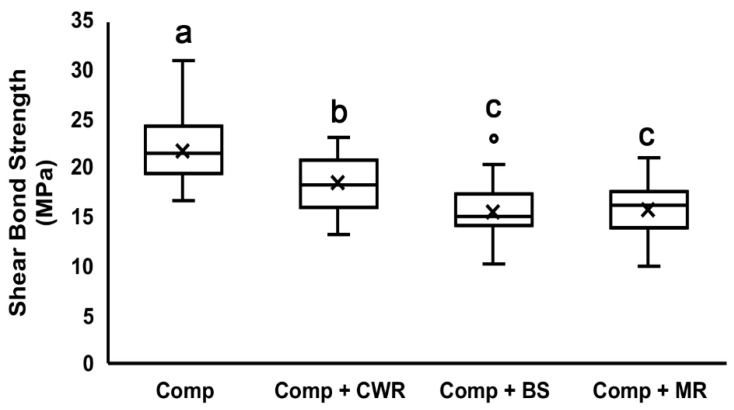
Shear bond test to the enamel surface. Composite mixed without or with different handling agents were bonded onto the enamel surface and subjected to the shear bond test. Different letters indicate significant differences between groups (*p* < 0.05). The ‘x’ in the box indicates the mean value of each group. Comp = Composite; CWR = Composite Wetting Resin; BS = Brush and Sculpt; and MR = Modeling Resin.

The results of the shear bond test to the enamel surface are shown as means ± standard deviations (Table 2) and a box plot (Figure 3). All tested CHAs demonstrated reductions in shear bond strength. Compared to the composite-only group (21.84 ± 3.49 MPa), the CWR group showed the least but a significant reduction (18.45 ± 2.98 MPa), followed by the BS group (15.66 ± 3.19 MPa) and MR group (15.84 ± 2.75 MPa). There was no statistically significant difference between the BS and MR groups (*p* = 0.998).

### 3.3. Effects of Resin Mixtures and Substrates on Shear Bond Strength

**Figure 4:**
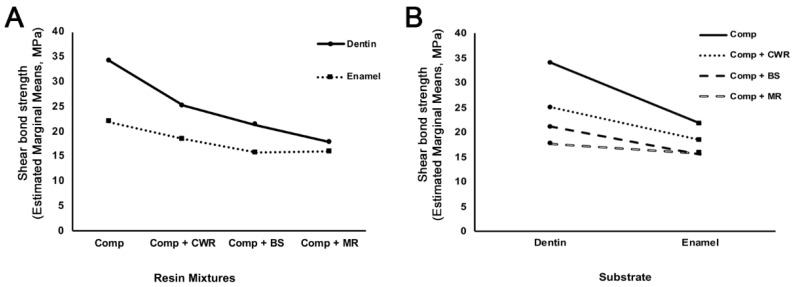
Effects of resin mixtures and substrates on shear bond strength. Profile graphs were plotted for the shear bond strengths for resin mixtures on substrates (**A**) or the substrates with the different resin mixtures (**B**).

Two-way ANOVA results are shown in Table 3. Statistically significant differences were found among resin mixtures (*p* < 0.001) and substrates (*p* < 0.001). Furthermore, statistically significant interactions between resin mixtures and substrates were found (*p* < 0.001). The profile graphs are shown in Figure 4. The shear bond strength to the enamel surface was less than that to the dentin surface. These data indicate that the shear bond strength between tooth and resin was affected by the CHAs used and the bonded substrate surfaces.

### 3.4. The Mode of Bonding Failure

**Figure 5:**
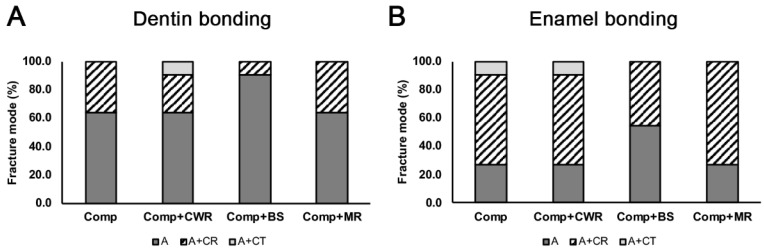
Mode of bond failure. The mode of bond failure was noted by observing the failed surface on the dentin (**A**) or enamel (**B**) surfaces. A = adhesive failure; A + CR = adhesive failure + cohesive (resin) failure; and A + CT = adhesive failure + cohesive (tooth) failure.

The results of the bonding failure mode are shown in Figure 5. Most of the bonding failure on the dentin surface was due to adhesive failure. On the other hand, a combination of adhesive and cohesive failure on the composite was the primary failure mode on the enamel surface. No complete cohesive failure was noted. BS displayed preferential adhesion failure mode on dentin and enamel surfaces among the groups.

## 4. Discussion

The disintegration of the bonding interface along with the subsequent marginal microleakage and recurrent caries are the primary modes of failure in modern composite resin restorations [22]. The shear bond strength is a widely accepted and representative parameter for the quality of the adhesive interface to the tooth [23,24]. Here, we examined the effect of composite-handling agents (CHAs) on the shear bond strength of composite and reported that the shear bond strength of composite onto the dentin and enamel substrates significantly decreased when mixed with the CHAs. To the best of our knowledge, this is the first study that examined the direct effect of CHAs on the composite shear bond strength.

Although material properties of composites have significantly improved over the past several decades, the handling property of composites, such as stickiness, remains poor due to their intrinsic viscosity. As a result, clinical outcomes of direct composite restorations are heavily dependent on dental practitioners’ techniques. Dental practitioners frequently use “wetting agents” to minimize voids and increase marginal adaptation to circumvent this technical issue. Alcohol and bonding adhesives were originally suggested as instrument lubricants, but numerous studies quickly disputed their use [25,26]. A single-component or self-etch adhesive system containing alcohol or acetone as a solvent is ruled inappropriate as an instrument lubricant due to the lack of evaporation when mixing with composite resin. For this reason, CHAs were developed with a 20–50% filler content to serve as a wetting agent. The question arises if the use of CHAs will also compromise the bonding interface and hybrid layer formed due to the inclusion of resinous matrices with less filler content, thereby negatively asserting the mechanical properties of the composite resin restoration.

Although CHAs are frequently utilized in the clinic, studies about the effect of CHAs on composites remain limited and controversial. One study examined the effect of modeling resin and thermocycling on the surface microhardness, surface roughness, and color of the tested composites. The authors found no significant changes, although they only utilized one modeling resin in their study [21]. Another similar study showed that the application of modeling resin, unlike resin monomer or primer, did not significantly alter the surface roughness of the final resin composites and maintained the best color stability after storage in coffee [19]. Barcellos et al. examined the effects of resinous monomer use as a modeling agent, primarily focusing on the cohesive strength of the composite, and found no alteration of the mechanical properties of the composite [27]. In contrast, a study by Munchow et al. showed that dental adhesives as modeling liquid of resin composites showed increased physical stability of the resin composites at the composite-to-composite interface [15]. Nevertheless, there is evidence suggesting that the usage of bonding adhesive as CHAs significantly increased the water uptake, which might lead to the decreased diametrical tensile strength of the composite [13,28]. Interestingly, there are no studies examining the effects of CHAs on the shear bond strength of composites.

Our study is unique in that we focused on the shear bond strength of composite mixed with CHAs at the dentin or enamel interface. Of all CHAs that are currently available, none of them were able to maintain a similar degree of shear bond strength at the dentin or enamel interface when compared to composite alone (Figure 4 and Table 2), suggesting that CHAs compromise composite bonding onto both the dentin and enamel substrates.

It is noteworthy that the degree of shear bond strength correlates with the amounts of filler contents in the CHAs (Figure 2 and Figure 3; Table 1). CWR achieved the highest shear bond strength among them, and it has the highest filler percentage, 45%. BS and MR have filler percentages of 36% and 20–40%, respectively, and their shear bond strengths were relatively lower. Generally, the shear bond strength decreases as the filler contents decreases [29,30]. Therefore, such a trend indicates that CHAs with a high filler content is desirable when used to handle composites.

In our study, shear bond strength on the dentin was higher than on the enamel (Figure 4). Although the shear bond strength of composite on the enamel is generally considered to be higher than that on the dentin [31,32], other reports demonstrated higher dentin bonding than enamel bonding [33]. Such mixed reports are attributed to the orientations of substrates’ cutting surfaces (e.g., the enamel rods and dentinal tubules) that primarily dictate bonding force. The composite bonding onto the enamel perpendicular to the enamel rods is stronger than those parallel to the enamel rods [34]. On the other hand, composite bonding onto the dentin perpendicular to the dentinal tubules is substantially weaker than those parallel to the dentinal tubules [35]. As our specimens were prepared sagittally where both enamel rods and dentinal tubules are expected to be aligned parallel to the composite bonding, our results are in line with previous findings. Clinically, however, bonding to enamel is expected to confer a better clinical outcome due to better marginal integrity and less hydrolytic breakdown [36].

In general, adhesive failure was more prominent in dentin bonding than enamel bonding. On the contrary, cohesive failure in the composite was seen more in the enamel bonding (Figure 5). Clinically, CHAs are more frequently used at the enamel margins to sculpt and adapt the composite restorations at the cavosurface margins. Given that CHAs may possibly dilute the original compositions of the composites, cohesive failure in the composites suggests that CHA-diluted composites may be subjected to failure and microleakages at the margins from long-term use of the restored tooth with heavy mastications and occlusions. The aging effects of CHA-diluted composites at the margins warrant further examinations.

There are several limitations to this study. First, this study is in vitro in nature that primarily focuses on the shear bond strength only. Second, we mixed the composites with the sculpting resins at a 1:1 weight ratio. This mixture ratio is based on our assumption that the amount of composite-handling resins while packing or adapting the composite at the margins is significant such that it may ultimately result in 50% dilution at the interface. This is particularly true considering the popular practice of incremental layering technique, where the last layer of composite resin placed at the margin may be heavily mixed with the CHA to improve adaptation. Due to the large variations in the amount of CHAs used among dental practitioners, it would be interesting to examine different degrees of dilution factors with CHAs on the shear bonding strength. Third, clinically, composite restorations are affected by various stresses, including compressive, flexural, tensile, or thermal stresses. Aging conditions such as thermocycling and/or fatigue loading conditions should be considered to obtain more clinically relevant results. Fourth, the combination with various base resins aside from Z250 is needed to verify the effects of CHAs. Therefore, additional studies are needed to thoroughly define the effect of composite-handling agents at the margins. Based on this study, our null hypothesis—that there will be no changes in the shear bond strength when mixed with composite-handling agents—is rejected. Our findings imply that composite-handling agents may weaken the shear bond strength of composite resin at the margin where CHA is applied, compromising overall marginal integrity. As such, the amount of CHAs should be minimized to prevent marginal leakage secondary to the failure of marginal integrity if the use of CHAs is inevitable.

## Figures and Tables

**Table 1:** Composite-handling agents used in this study.

Products	Manufacturer	Filler (%)	Filler Material	Organic Matrix
Composite Wetting Resin **(CWR)**	Ultradent	45%	Undisclosed	DUDMA, BHT, TEGDMA
Brush and Sculpt **(BS)**	Cosmedent	36%	0.04 µm silicon dioxide	UDMA, Bis-GMA, 1,4-Butanediol dimethacrylate
Modeling Resin **(MR)**	BISCO	20–40%	Amorphous silica	UDMA, Bis-GMA, TEGDMA, ethoxylated Bis A dimethacrylate

Abbreviations: DUDMA = diurethane dimethacrylate; BHT = butylated hydroxytoluene; TEGDMA = triethylene glycol dimethacrylate; UDMA = urethane dimethacrylate; and Bis-GMA = bisphenol A-glycidyl methacrylate.

**Table 2:** Shear Bond Strength results (MPa).

Group	Dentin (Mean ± STD)	Enamel (Mean ± STD)
Comp	34.16 ± 3.65 ^a^	21.84 ± 3.49 ^a^
Comp + CWR	25.09 ± 3.10 ^b^	18.45 ± 2.98 ^b^
Comp + BS	21.24 ± 3.37 ^c^	15.66 ± 3.19 ^c^
Comp + MR	17.73 ± 3.26 ^d^	15.84 ± 2.75 ^c^

Different letters indicate significant differences between groups (*p* < 0.05) in either dentin or enamel group, respectively.

**Table 3:** Analysis of two-way ANOVA.

Source	Type III Sum of Squares	Df	Mean Square	F	*p*
Corrected Model	5251.171 ^a^	7	750.167	71.685	<0.001
Intercept	72,284.004	1	72,284.004	6907.367	<0.001
Resin Mixtures	2943.575	3	981.192	93.761	<0.001
Substrate	1747.684	1	1747.684	167.006	<0.001
Resin Mixtures * Substrates	559.912	3	186.637	17.835	<0.001
Error	1590.645	152	10.465		
Total	79,125.820	160			
Corrected Total	6841.816	159			

^a^ R Squared = 0.768 (Adjusted R Squared = 0.757).

## Data Availability

Not applicable.
